# Home exercise improves the quality of sleep and daytime sleepiness of elderlies: a randomized controlled trial

**DOI:** 10.1186/s40248-017-0114-3

**Published:** 2018-01-15

**Authors:** Glauber Sá Brandão, Glaucia Sá Brandão Freitas Gomes, Glaudson Sá Brandão, Antônia A. Callou Sampaio, Claudio F. Donner, Luis V. F. Oliveira, Aquiles Assunção Camelier

**Affiliations:** 1 Bahiana School of Medicine and Public Health, Salvador, BA Brazil; 2 Department of Education (DEDC-VII), University of the State of Bahia – UNEB, Rua Visconde do Rio Branco 162, Centro, Senhor do Bonfim, BA CEP 48970-000 Brazil; 3 IMAIS – Diagnostic and Specialty Clinic, Senhor do Bonfim, BA Brazil; 4 Mondo Medico, Multidisciplinary & Rehabilitation Outpatient Clinic, Borgomanero, NO Italy; 5 Medical School, University Center of Anapolis – UniEVANGELICA, Anapolis, GO Brazil; 6 Department of Life Sciences (DCV), University of the State of Bahia – UNEB, Salvador, BA Brazil

**Keywords:** Community elderly, Exercise, Sleep

## Abstract

**Background:**

Aging causes physiological changes which affect the quality of sleep. Supervised physical exercise is an important therapeutic resource to improve the sleep of the elderlies, however there is a low adherence to those type of programs, so it is necessary to implement an exercise program which is feasible and effective. The study aimed to test the hypothesis that a semi-supervised home exercise program, improves sleep quality and daytime sleepiness of elderlies of the community who present poor sleep quality.

**Methods:**

This was a randomized controlled trial study, conducted from May to September 2017, in Northeastern Brazil, with elderlies of the community aging 60 years old or older, sedentary, with lower scores or equal to 5 at the Pittsburgh Sleep Quality Index (PSQI) and without cognitive decline. From one hundred ninety-one potential participants twenty-eight refused to participate, therefore, one hundred thirty-one (mean age 68 ± 7 years), and 88% female, were randomly assigned to an intervention group - IG (home exercise and sleep hygiene, *n* = 65) and a control group - CG (sleep hygiene only, *n* = 66). Sleep assessment tools were used: PSQI, Epworth sleepiness scale (ESS) and clinical questionnaire of Berlin. The level of physical activity has been assessed by means of International Physical Activity Questionnaire adapted for the elderly (IPAQ) and Mini-Mental State Examination for cognitive decline. All participants were assessed before and after the 12-week intervention period and, also, the assessors were blind.

**Results:**

The IG showed significant improvement in quality of sleep with a mean reduction of 4.9 ± 2.7 points in the overall PSQI (*p* < 0.01) and in all its 7 components of evaluation (*p* < 0.05), and improvement of secondary endpoint, daytime sleepiness, a decline of 2.8 ± 2.2 points in the ESS (p < 0.01).

**Conclusion:**

Our results suggest that semi-supervised home exercise is effective in improving the quality of sleep and self-referred daytime sleepiness of sedentary elderlies of the community who presented sleep disorders.

**Trial registration:**

Ensaiosclinicos.gov.br process number: RBR-3cqzfy.

## Background

The natural process of human aging causes important organic changes, resulting in mental and physical alterations which involve quality of life of the elderlies [1, 2]. Among the main health problems observed in elderly population, with the incidence increasing proportionally with age, sleep disorders are among the most prevalent, and more than 50% of the elderlies complain about their sleep quality [3–6].

For this specific population, the pharmacologic therapeutic approach still is the most commonly used to treat sleep disorders [7]. However, the frequent use of sleeping pills alter sleep architecture and they are associated to several adverse effects such as sedation, excessive drowsiness daytime, increased risk of falling and higher functional dependency [7, 8], which usually are frequently observed in elderlies [9]. Therefore, alternative strategies are needed to improve the quality of sleep in this population. The physical exercise of mild to moderate intensity, presents positive results in the sleep quality of elderlies and it is recommended as one of the key features of preventive and therapeutic non-pharmacologic intervention [10, 11]. However, despite scientific evidence demonstrating the benefits of physical exercise in health and quality of life, yet there is little adhesion to this strategy by the elderlies [12].

The difficulty in the transfer to the location of the exercises, schedule conflict with household tasks, conditioning difference between participants and the limited supply of free programs associated to the low economic power of this population, are possible explanations for the low adhesion [13, 14]. These difficulties presented by the majority of the elderlies in supervised exercises, associated with the benefits of their practice, encourage the development of other feasible programs which allow greater adherence. In a recent systematic review, the authors concluded that home-based exercise programs show better adherence when compared to group programs [15]. However, according to our knowledge, a single reference was located on the practice of semi-supervised home exercise and quality of sleep of elderlies [16].

In view of the foregoing and considering this knowledge gap, the present study aimed to test the hypothesis that an alternative program of activities, through physical exercise performed at home, semi-supervised, easy to perform and low cost, improves the perception of sleep quality, and excessive daytime sleepiness of sedentary elderly people in the community who present sleep disorders.

## Methods

### Study design

It is an analytical, experimental, randomized, controlled, single blind study conducted according to the CONSORT stands for Consolidated Standards of Reporting Trials [17].

### Participants

The study involved elderly aged 60 years or older, living in the city of Senhor do Bonfim - BA in the Brazil Northeastern region, in the period from May to September 2017. The recruitment occurred throughout the community, initially through local newspapers, radio, religious centers, encounter groups of elderlies, senior residence, association neighborhoods and in the senior project developed by the municipal government. Inclusion criteria were: absence of regular exercise in the last three months before the beginning of the study and score more than or equal to 5 in the Sleep Quality Index Pittsburgh (PSQI-BR) [18]. We excluded the subjects presenting cognitive decline according to the Mini-Mental State Examination [19] or performing treatment for sleep disorders (including the use of sleep medications) or presenting any clinical condition that would contraindicate the performance of physical activity, identified through a medical and physiotherapic evaluation.

The study was approved by the Ethics Committee on Research Involving Human Subjects of Bahia School of Medicine and Public Health and all participants agreed to participate and signed the free and informed consent. This trial is registered in ensaiosclinicos.gov.br (identifier: RBR-3cqzfy).

After meeting the eligibility criteria, the subjects were identified in consecutive order of entry into the study and were then randomly allocated according to a sequence of random numbers generated by the Research Randomizer (https://www.randomizer.org/). This randomization was of the closed type, with concealment of the allocation, generating two groups, being one the control group (CG) and the other the intervention group (IG).

All the elderlies involved in the present study participated in a 40-min presentation with explanations about the experimental procedure and they received educational leaflets containing guidelines on life habits related to feeding, hydration and sleep hygiene. The IG participants were informed that they should follow lifestyle guidelines and conduct a home physical exercise program. For that, they participated in a theoretical-practical training aimed at the adequate accomplishment of the proposed exercises and received a primer developed by the researchers, with illustrated and written guidelines on the accomplishment of the exercises, as well as a journal to record the weekly frequency of its accomplishment. The researchers, after making sure that the subjects could perform all the exercises adequately, guided the family members to help and stimulate the practice and encouraged the elderlies to call in situations of problems or doubts. The participants of the CG were informed that they should only continue with their activities of daily living and follow the guidelines related to their habits of life.

Previously, before the beginning of the study, there were systematic trainings of assistants, five students of the physiotherapy course, exclusively for the evaluation, and ten other assistants, also students of the physiotherapy course, for home monitoring of the elderlies, five visited the subjects of the IG and the other five visited the elderlies of the CG. The distribution of the number of subjects to be evaluated and the number of domiciles to be visited was done in an equivalent way among the research assistants.

## Evaluation protocol and procedures

### Assessments

The evaluations were performed before and after the intervention period, by a doctor, a physiotherapist and the assistants, in which, the elderly received standard verbal instructions regarding the procedures and they were evaluated individually in an appropriate room. The researchers in charge of the data analysis were blinded to groups of subjects, avoiding possible biases.

A general physical and clinical evaluation was performed, with collection of socioeconomic, demographic, anthropometric, and self-referenced morbidities data. The quality of sleep, excessive daytime sleepiness, the risk of obstructive sleep apnea syndrome and the level of physical activity were also evaluated.

The primary endpoint of the present study was the quality of self-reported sleep, verified through the Pittsburgh Sleep Quality Index (PSQI). The PSQI was developed in 1989 by Buysse DJ [20] and validated for the Brazilian population [18]. This instrument allows an assessment of the quality of sleep, categorizing the subjects in good or bad sleepers.

The secondary endpoint was presence of excessive daytime sleepiness recorded using the Epworth Sleepiness Scale (ESS), validated in Brazil [21].

In order to assess the potential risk of the presence of obstructive sleep apnea (OSA) we used the Berlin clinical questionnaire [22]. This instrument considers a high risk for OSA, when two or more categories present positive score and when it shows none or only one category with a positive score the risk for OSA is low.

In the evaluation of the anthropometric variables, Body Mass Index (BMI) was calculated from the weight in kilograms divided by the height in squared meter.

The level of physical activity was assessed using the International Physical Activity Questionnaire (IPAQ) adapted for the elderly [23]. It is an instrument that allows estimating the weekly energy expenditure of physical activities related to labor, transportation, domestic tasks and leisure, performed for at least 10 continuous minutes with moderate and/or vigorous intensity during a normal/usual week. This variable was dichotomized, and those who performed less than 150 min per week of moderate and/or vigorous physical activity were considered non-active and active those who performed more than 150 min per week.

## Intervention

The home exercise program was based on American College of Sports Medicine recommendations for exercise and physical activity with the elderly [24]. The exercise program was composed by a combination of aerobic exercises, muscle strengthening, balance, coordination and flexibility, prioritizing exercises involving large muscle groups. The protocol lasted 12 consecutive weeks, with minimum frequency of three weekly sessions, predicted time of 40 min and performing 2–3 sets with 5–15 repetitions for each exercise, a target effort rate of 13–15 (“a bit difficult” to “difficult”) in the range of perceived exertion Borg 6–20 points [25], being the exercise performed in the convenience turn chosen for the elderly. The exercises were performed individually at home by each participant, having no supervision during implementation; however, receiving guidance through home visits every 15 days. The subjects were instructed to increase exercise intensity, using as parameter the Borg range and proportional form for their implementation capacity, assessed by research assistants in each visit.

The exercises were conducted by using the subject’s own weight body and with the help of some low-cost equipment (e.g., recyclable plastic bottles to demarcate the signaling route rods and weights of 1 and 2 kg for implementing the resistance exercises). The protocol was performed as follow:

- Warm up exercises - Free-active exercises involving upper and lower limbs and movements of rotation of the shoulders associated with breathing exercises;
- Aerobic exercises - Displacement of a stick with both hands, starting from the knees up over the head and returning to the knees. Ambulation exercises with alternating bending of the thighs and approaching the knees to the hands on the opposite side;
- Endurance Exercises - Upper limbs departing from the position with the extended elbow and the hand resting on the opposite thigh, moving the whole limb diagonally upward and then returning the hand to the thigh. For the lower limbs, squatting exercises, starting from the sitting position on a chair and with arms crossed in front of the body, lifting to the orthostatic position and then returning to the sitting position;
- Balance and coordination exercises - Walk on a straight line on the ground and walk from queued obstacles with progressively smaller distances. When possible, the exercise evolved and the walk was performed by placing the heel of one foot on the toes of the other foot (standing foot).

Note: To ensure safety, these exercises were performed close to fixed furniture in the house, making it possible to lean when needed.

- Stretching Exercises - Starting from a sitting position on the bed or a chair, with your knees in extension trying to reach the tip of the feet; from the sitting position in a chair and with the feet resting on the ground, perform the rotation of the trunk to one side and elevation of the upper limb, on the same side, above the head, stretching as high as possible.

During the period of 12 consecutive weeks, the subjects of both groups received periodic home visits with the purpose of continuing with the guidelines on lifestyle and encouraging the adherence to the program; however the IG, in addition to guidelines on habits of life, received specific follow up regarding exercise practice and assistance to possible adverse events. After three months, the participants of both groups were re-evaluated and at the end of protocol the elderlies of the IG were encouraged to continue with the exercises, while the CG was made available the follow up of the home exercises for the same period performed with the IG.

The adherence to the exercise was verified through the weekly records filled out by the subjects themselves, with the help of family members and certified by the assistants during the home visits.

## Statistical analysis

The sample size calculation based on recent studies [16] demonstrated that it would take 63 participants per group to obtain a statistical power of 80% in the detection of 2-point difference with 5% alpha, considering a standard deviation estimated 4 points. The principle of intention-to-treat analysis was respected and for the missing data the sensitivity analysis was performed through simple imputation using the mean of the variables. To detect if randomization produced comparable groups, the characteristics of both groups were compared before the intervention using the Student’s t-test for independent samples in relation to the numerical variables and the Pearson chi-square test for categorical variables. To test the normality of the data, the histogram, mean and median, standard deviation, skewness and kurtosis were analyzed and the Shapiro-Wilk normality test was used for confirmation. Due to the normal distribution of variables, parametric statistics were used, and intra-group comparisons were performed using Student’s t-test for paired samples.

A subgroup analysis of extracts of age was pre-specified in the study protocol, as it was done with more than two groups that had parametric distribution, we used one-way ANOVA. The significance level set to for the analyses was set at *p* < 0.05 and statistical procedures were analyzed and processed in the Statistical Package of Social Sciences (SPSS 21.0). IBM SPSS version 21 (IBM, Armonk, NY).

## Results

**Fig. 1:**
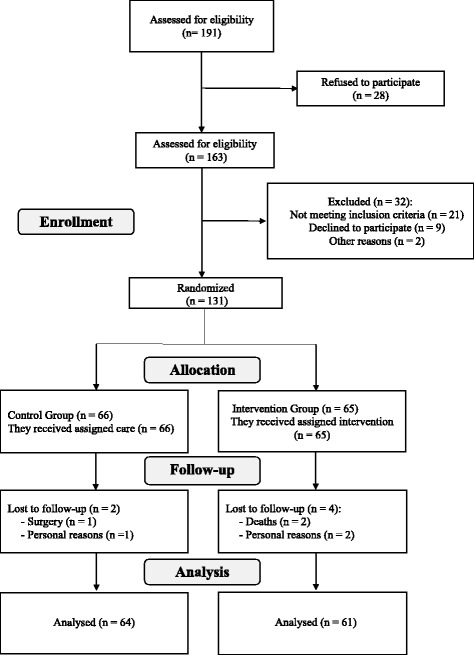
Flowchart of the study

One hundred and ninety-one potential elderlies candidates were screened by phone. 28 elderlies refused to participate in the examination, therefore 163 elderlies were enrolled in the study, of which 32 were excluded according to the eligibility criteria. 131 subjects were randomized elected, and thus constituted the IG with 65 participants and the CG with 66 subjects. During the study, there were follow up losses of 2 participants of the CG and 4 of the IG, allowing to 125 elderlies to conclude the follow up. A summary of the randomization, participant flow, and follow up losses during the trial are shown in Fig. 1.

The patients involved in the present study showed a predominance of females (88%); mean age 68 ± 7 years and a mean BMI of 27.3 ± 4, indicative of pre-obesity. The socioeconomic profile showed a predominance of low income in relation to the monthly minimum wage (84.8% ≤ 2 minimum wage) and low schooling (86.3% ≤ 3 years of study). 87% of the elderlies presented a level of physical activity considered active (IPAQ > 150 min per week).

Most of the subjects lived with their families (88%), were nonsmokers (91%), non users of alcoholic beverages (88%) and presented as main self-reported morbidities anxiety, arthrosis, hypertension and diabetes. 38.2% of the subjects are considered to be at high risk for OSA because they present a positive score in two or more categories of the Berlin Clinical Questionnaire. The mean PSQI score was 11.2 ± 3.2 and ESS score was 8.6 ± 2.8. The Table 1 presents the characteristics of the two groups, at baseline, with no statistically significant difference.

**Table 1:** Baseline characteristics by group

Variables	Control group (*n* = 64)	Intervention group (*n* = 61)	*p*
Age (years)	69.9 ± 6.7	69.8 ± 7.4	0.76
Gender (% women)	84.4	91.8	0.23
BMI (Kg/m^2^)	27.7 ± 4.7	27.6 ± 4.1	0.75
Waist Circumference (cm)	93 ± 10	93 ± 10	0.76
Number of self-reported morbidities	1.9 ± 1.4	1.8 ± 1.5	0.63
Per capita income (% ≤ 2 minimum wages)	82.3	85.7	0.86
Education (% ≤ 3 years of study)	75	75.9	0.54
Housing (% live with relatives)	67	71	0.85
Pittsburgh Sleep Quality Index	11.4 ± 3	11 ± 3.4	0.55
Epworth Sleepiness Scale	8.5 ± 3	8.7 ± 3	0.14
Berlin Questionnaire (% high risk)	37	39	0.23
Physical Activity – IPAQ (% actives)	88	85	0.34

Data mean ± standard deviation or in (%); *n* = number of participants who completed the follow up. No significant difference was detected between groups (*p* > 0.05)

The exercise average frequency over the entire 12-week period was 4 ± 0.6 days per week, with a minimum of 3 days per week performed by 3 participants and a maximum of 6 days per week performed by 1 participant. All participants of the IG had 100% adherence to the exercises and there was no report on any type of njury related to the intervention program.

**Fig. 2:**
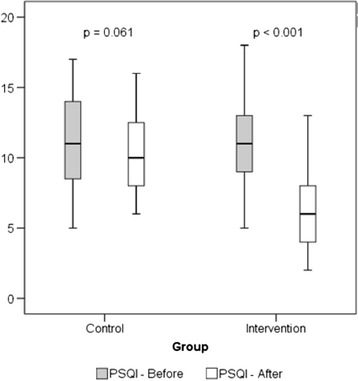
Comparison of quality of sleep (PSQI), before and after the intervention, in each group. T-test for paired samples

Figure 2 shows an analysis of the overall quality of sleep improvement, by comparing the global PSQI score before and after the intervention in each group, demonstrating that the improvement of sleep quality was statistically significant only in the IG with a mean reduction of 4.9 ± 2.7 points (*p* < 0.001) compared to 0.7 ± 2.8 in the CG (*p* = 0.061). Subgroup analysis by ANOVA, performed in the IG, showed that the improvement in overall sleep quality did not present a significant difference when comparing the ages 60 to 69, 70 to79 and ≥80-year-old, *p* = 0.15.

Table 2 shows the sleep evaluation components constituting the PSQI, demonstrating that only the GI showed statistically significant improvements in all components.

**Table 2:** Delta of mean PSQI components related to pre- and post-intervention for each group and *p* of the difference

Variables	Group	Basal moment	After 12 weeks of intervention	Delta	p*
Subjective sleep quality	CG	1.9 ± 0.7	1.8 ± 0.6	0.1	0.16
	IG	1.8 ± 0.7	1.1 ± 0.6	0.7	< 0.01
Sleep latency	CG	2.0 ± 0.9	1.7 ± 0.8	0.2	0.08
	IG	1.9 ± 1.0	1 ± 0.9	0.9	<0.01
Duration of the sleep	CG	1.9 ± 0.9	1.6 ± 0.8	0.3	0.11
	IG	2.1 ± 1	1.2 ± 0.8	0.8	<0.01
Usual sleep efficiency	CG	2 ± 0.9	1.6 ± 1.1	0.4	0.06
	IG	1.6 ± 1.0	0.7 ± 0.9	0.9	<0.01
Sleep Disorders	CG	2 ± 0.6	1.9 ± 0.7	0.1	0.41
	IG	1.9 ± 0.6	1.2 ± 0.5	0.6	<0.01
Use of sleeping medicines	CG	0.3 ± 0.4	0.2 ± 0.4	0.0	0.32
	IG	0.2 ± 0.4	0.1 ± 0.3	0.1	0.04
Dysfunction during the day	CG	1.6 ± 1.2	1.7 ± 1	−0.1	0.57
	IG	1.7 ± 1.1	1.1 ± 0.8	0.6	<0.01

Data mean ± standard deviation

*t-test for non-paired samples (*p* < 0.05)

**Fig. 3:**
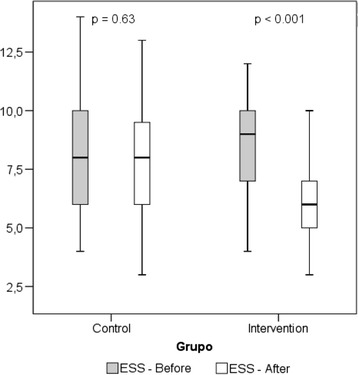
Comparison of improving excessive daytime sleepiness (ESS), before and after the intervention in each group. T test for paired samples

The evaluation of the presence of daytime sleepiness in the groups, through ESS before and after the intervention, in each group, found that only the IG presented a statistically significant reduction, with a mean variation of 2.8 ± 2.2 points (*p* < 0.001) compared to 0.14 ± 2.3 (*p* = 0.63) in the CG (Fig. 3).

## Discussion

The results of the present study showed that sedentary elderlies who present sleep disorders while participating in a semi-supervised home physical exercise program presented significant improvement in sleep quality and reduction of self-reported excessive daytime sleepiness, verified in a non-objective way through questionnaires, being consistent with the hypotheses of the study and configuring a clinically relevant change.

Our results are consistent with previous studies which demonstrated the efficacy of physical exercise programs in sleep quality and in the reduction of daytime sleepiness in the elderlies [10, 11, 26, 27]. However, these studies used exercise programs with professional supervision during their execution, in addition to being performed in training and/or rehabilitation centers, which is a limiting factor for the participation of the elderlies, as they present difficulties in relation to the transfer [14, 28, 29].

However, in the present study, a home physical exercise program was applied, with only one-week supervision, easy to apply and low cost, which proved to be safe and feasible, since no injuries related to training were observed as well as high frequency rates (mean of 4 ± 0.6 days per week) and small loss of follow up (4.5%).

It has already been demonstrated in the literature that physical exercise performed at home may result in important health benefits for the elderly and, because it is appreciated by the elderly [29], there is still greater adherence and continuity after the end of the program [15, 30].

However, the knowledge regarding the effects of home exercises on sleep quality of the elderly is still very incipient. In the present study, the elderly who participated in the home exercise program during the 12-week period showed significant improvement over the primary endpoint, self-reported quality of sleep, represented by a reduction of 4.9 points in the overall PSQI score (Fig. 2). The significant reduction in all components of PSQI assessment, especially in relation to sleep latency, sleep duration and habitual sleep efficiency (Table 2), characterizes a relevant clinical improvement, being the results consistent with the study performed by Chen et al. [16]. The authors used a similar methodology to demonstrate the effectiveness of home physical exercise, by practicing the “Baduanjin” exercise technique in the self-reported quality of sleep. In that study, 56 elderly people (mean age 71.7 ± 8.1) were randomized, and the IG performed the “Baduajin” exercise in their households and the CG did not perform any specific activity. The IG received a videotape, an educational booklet with pictures about the performance of the exercises, and they were instructed to perform 30 min of exercise 3 times a week for 12 weeks and received telephone follow up twice a week. After this period, the IG showed a statistically significant improvement when compared to the CG in relation to the overall PSQI score and in 5 of the 7 evaluation components, similar to the results found in the present study.

The secondary endpoint, excessive daytime sleepiness, recognized as an important public health problem [31], was evaluated through the ESS demonstrating that the elderlies who practiced physical exercise presented a significant reduction of excessive daytime sleepiness, when compared to the CG, in which the reduction was not significant (Fig. 3), being consistent with the results of other studies which used supervised exercises of Yoga [27] and Tai Chi [32], which also demonstrated a significant reduction of excessive daytime sleepiness in the exercise group, using of the same rating scale.

The analysis of variance performed in the age subgroups has showed a significant improvement in sleep quality, however there was no significant difference, demonstrating that the home exercise program was effective in the different age groups of the elderly involved in the present study.

One of the strengths of our study is that, after random allocation of subjects, both groups were periodically given the same orientations and stimuli in relation to life habits such as feeding, hydration and sleep hygiene. These positive results presented in our study are also related to the fact that, in addition to the program being carried out at home and composed of dynamic and easy to be performed physical exercises, the elderlies received visits every fortnight and then they were encouraged and oriented, by the research assistants and their relatives, in relation to practice of the exercises, contributing to the high rate of adherence and small loss of the participants. We also highlight the lack of reports of adverse events during the exercise period.

Previous studies have suggested that a direct contact with participants via phone, internet or personal visits increases the adherence to home exercise programs [29, 30]. Follow up losses did not interfere in the results, since they occurred at random, their characteristics are homogeneous both in terms of quantity and quality of losses, since they corresponded to less than 10% of the total sample and presented reasons similar in both groups, besides the characteristics of those who remained in the study are comparable to those who did not remain.

All these results should be interpreted taking into account some limitations of the present study. Firstly, recruitment was performed with elderlies of the community with self-reported sleep disorders, however the results may be more clinically reliable if the participants are clinically diagnosed for the disorders. There is a predominance of females in the sample, which is justified by the feminization of the elderly population [33, 34] and the prevalence of sleep disorders in the female elderly [35], however the randomization generated equivalent distribution between groups. The inability to blind participants in relation to the intervention may have been tempered by the fact that each group (CG and IG) was accompanied by different assistants, minimizing the excitement bias applied by the IG assistants during home visits. The monitoring of the frequency of the exercises was self-reported, however to increase the reliability of that information, in addition to monitoring the frequency register during visits, family members were recruited to assist in collecting information. The quality of sleep was measured in a non-objective instrument, by means of validated questionnaire, that represented as perceived by the individual.

Future studies are needed to evaluate the effects of semi-supervised home-based exercise programs with long follow up periods and using objective strategies for assessing sleep quality, and to monitor and to quantify treatment efficacy, making outcomes clinically more reliable.

## Conclusions

The results of the present study suggest that the regular practice of semi-supervised physical exercises at home is effective in improving the self-reported quality of sleep and the reduction of daytime sleepiness in sedentary elderly people with sleep disorders in various age groups of the elderly population. Therefore, it may be considered as a therapeutic, non-pharmacological, easy-to-implement and safe resource for improving the quality of sleep of the elderlies.

## Abbreviations

BMI: body mass index
ESS: Epworth Sleepiness Scale
IPAQ: International Questionnaire of Physical Activity
OSA: Obstructive Sleep Apnea
PSQI: Pittsburgh Sleep Quality Index
